# Antenatal Depression is Not Associated with Low-Birth Weight: A Study from Urban Pakistan

**DOI:** 10.3389/fpsyt.2014.00175

**Published:** 2014-12-10

**Authors:** Nusrat Husain, Tariq Munshi, Farhat Jafri, Meher Husain, Asia Parveen, Qamar Saeed, Barbara Tomenson, Farooq Naeem, Nasim Chaudhry

**Affiliations:** ^1^Institute of Brain, Behavior and Mental Health, University of Manchester, Manchester, UK; ^2^Department of Psychiatry, Queen’s University, Kingston, ON, Canada; ^3^Department of Community Medicine, Medical and Dental College, Karachi, Pakistan; ^4^Harvey House Social Enterprises, Lancaster, UK; ^5^Pakistan Institute of Learning and Living, Karachi, Pakistan; ^6^Chiniot Maternity Home, Karachi, Pakistan; ^7^Biostatistics Unit, Institute of Population Health, University of Manchester, Manchester, UK

**Keywords:** low-birth weight, mental health, depression, prenatal development, developing country, antenatal depression, prenatal depression, low and middle income countries

## Abstract

**Background:** Low-birth weight (LBW) (<2500 g) is considered to be a leading cause of cognitive impairment and physical disabilities in children. Incidence of LBW in South Asia has been reported to be as high as 33%. We investigated the association between antenatal depression and LBW in an urban community, in Karachi, Pakistan.

**Methods:** A total of 1357 pregnant women in their third trimester were recruited into the study. They were screened for antenatal depression with Edinburgh postnatal depression scale. Self-reporting questionnaire was also used to measure psychological distress. Birth weights of 763 newborns were obtained from the hospital records.

**Results:** We did not find a significant association between antenatal depression and LBW (odds ratio 0.881, 95%CI 0.732–1.060) in mothers attending a charity run hospital in an urban setting in Pakistan.

**Conclusion:** Antenatal depression is not associated with LBW in this urban population in Pakistan. However, the prevalence of depression is high during pregnancy. There is a need to develop culturally adapted psychosocial interventions to address the high rates of depression for this population group.

## Introduction

Low-birth weight (LBW) is a major public health problem in low and middle income countries (LAMIC). It is associated, not only with increased risk of mortality but is also one of the leading causes of peri-natal morbidity and neuro-developmental impairments in newborn babies (1). LBW has been defined by the World Health Organization (WHO) as “weight at birth of <2500 g (5.5 Ibs).” This is based on epidemiological observations that infants weighing <2500 g are approximately 20 times more likely to die than heavier babies (2).

More common in developing than developed countries, a birth weight below 2500 g contributes to a range of poor health outcomes (3). Main complications found in LBW babies are asphyxia, hypothermia, bronchopulmonary dysplasia, hypoglycemia, hyperbilirubinemia, infection, anemia, retinopathy, and dehydration (4). LBW is closely associated with fetal mortality and morbidity, inherited growth and cognitive development abnormalities, and chronic diseases later in life. At the population level, the proportion of babies with a LBW is an indicator of a multifaceted public health problem that includes long-term maternal malnutrition, ill health, hard work, and poor health care in pregnancy. On an individual basis, LBW is an important predictor of new born health and survival and is associated with higher risk of infant and childhood mortality (5)

The incidence of LBW is estimated to be around 16%, worldwide, and 33% from the South Asia (6). Research findings from the developed world regarding the association between antenatal depression and LBW are inconsistent, as some studies report positive association, (7–9), while others reported a negative association (10–13). Studies from the LAMIC also report mixed results. Although some studies from South Asian countries report a negative impact of antenatal depression on infant birth weight (14–16), one recent study from Ethiopia did not confirm a such association (17).

In the Western world, between 6.5 and 12.9% mothers experience depression around child birth (18). The rates are much higher in South Asian mothers (19–21). Earlier studies from Pakistan, reporting association between maternal depression and the LBW were conducted in rural population (14, 22). The pace of urbanization in Pakistan is very high, and it is important to determine whether similar factors are operating in the urban population. It is important to explore this association to confirm the findings from previous studies, conducted in rural population in Pakistan, and elsewhere. This information can then be used to further refine and retest interventions to help mothers with antenatal depression. This has significant implications in resource utilization in LAMIC. We are not aware of published work from urban Pakistan in this area. The aim of this study was to look at the association between antenatal depression and LBW, using a large hospital based sample in an urban population of a low-income country.

### Hypothesis

Our hypothesis was that antenatal depression is associated with LBW.

## Materials and Methods

This cohort study was conducted in Karachi from March 2004 to April 2005. The study sample was drawn from the antenatal clinic of a charitable Trust, called the “Chiniot Maternity Home and child care center,” situated in the Central District in Karachi, which provided services for the lower middle class population. Consecutive patients visiting the antenatal clinic in their third trimester were recruited by a female researcher. Those who met the inclusion criteria were asked for written informed consent. A total of 1403 mothers were invited to take part in the study of whom, 46 refused to participate (response rate 96.7%).

### Study area

Pakistan is a low-middle income country, with a population of more than 180 million. The literacy rate among women is only 35%. The infant mortality rate is about 67 per 1000 live births. Being a developing country, most of its population (60%) earns below Rs 168 (US$2) per day. This study was conducted between March, 2004 to April 2005, at a 34-bed maternity and child care center. The Chiniot Maternity and Child Care Centre is located in the central district in Karachi, Pakistan. It is well known for its charity based low-cost nominal pricing policy with high standards of care and management.

### Data collection

The personal information questionnaire (PIQ) was used to gather detailed personal information. Data were also collected on parents’ educational status, household income, and whether the mother had a problem with a previous delivery. All expecting mothers in their third trimester of the pregnancy were interviewed using an Urdu version of the Edinburgh postnatal depression scale (EPDS). Newborns’ weights were obtained from the hospital records. A cut-off of 2500 g or less was used for LBW in neonates.

### Self-reporting questionnaire

To assess psychological distress during pregnancy and after childbirth, self-reporting questionnaire (SRQ-20) (23) was administered. This questionnaire consists of 20 items, and, each item has a yes/no answer. Therefore, each item is scored as 0 or 1. The maximum score is 20. The SRQ inquires about the symptoms, over the past 30 days. The higher the SRQ score, the greater the distress. A cut-off score of seven or more on the SRQ-20 is used in this study to indicate clinically significant psychological distress. Since, most studies from developing countries have used this cut-off point (23). SRQ has been validated in Pakistan in our previous study (24).

### Edinburgh postnatal depression scale

A validated 10 item EPDS (25) was used to screen for antenatal and postnatal depression. The EPDS was originally designed and validated for postnatal depression. However, it has also been found to be effective for the detection of antenatal depression (26, 27). The EPDS consists of 10 self reported items, rated on a scale of 0–3. A score of ≥12 is used to distinguish cases of depression from non-cases. The higher the EPDS score, the greater is the severity of depression. We have used EPDS in our earlier studies (20), and it has been validated with the Pakistani population (21).

### Statistical analysis

The data were analyzed using SPSS version 20. In univariate analyses, the effect of potential risk factors on LBW (under 2500 g) was tested using Fisher’s exact test for dichotomous variables, Yates’ continuity corrected Chi-squared test for other categorical variables, and the *t*-test for the mother’s age. Numbers and percentages within birth weight group are presented as categorical variables. Antenatal depression, using both EPDS and SRQ scores was categorized into four quartile groups, and multiple logistic regression was used to calculate the association between each measure and LBW of neonates. Odds ratios and 95% confidence intervals are presented, both unadjusted and after controlling for confounding effects of possible risk factors, maternal education, maternal age, income, paternal education and whether there was any problem with the previous delivery.

## Results

One thousand three hundred and fifty-seven women completed interviews at the baseline. This paper concerns the cohort of 763 (56.2%) mothers for whom we obtained the newborns birth weight. These mothers were significantly better educated and also significantly more likely to have a problem with their previous delivery than the remainder of the mothers. Birth weights ranged from 1400 to 4500 g (mean = 2904 g, SD = 462), with 103 (13.5%) of the babies being under 2500 g. There were no significant differences in any of the potential risk factors measured between mothers of babies weighing 2500 g or more and mothers whose babies weighed under 2500 g at birth (Table 1). The mother’s mean age was similar in the two groups (birth weight over 2500 g, mother’s mean age = 31.8 years, SD = 5.2: birth weight under 2500 g, mother’s mean age = 31.8 years, SD = 5.3, *t* = 0.1, *p* = 0.88). Of the 183 mothers who were depressed at baseline (using the EPDS cut-off of 12 or more), 26 (14.2%) had a baby weighing under 2500 g, compared with 77 (13.3%) out of 580 mothers who were not depressed at baseline, Fisher’s exact *p* = 0.80. Of the 387 mothers who scored 7 or more on the SRQ, 53 (13.7%) had a baby weighing under 2500 g, compared with 50 (13.3%) out of 376 mothers who scored below 7, Fisher’s exact *p* = 0.92.

**Table 1:** **Characteristics of the study participants: those whose baby weighed 2500 g or more at birth compared with those whose baby was under 2500 g**.

Potential risk factor	Whole group (*n* = 763)		Birthweight 2500 g or more (*n* = 660)		Birthweight <2500 g (*n* = 103)		Sig^a^
	*N*	%	*N*	%	*N*	%	*p*
Monthly income: <6000 Rs	266	34.9	235	35.6	31	30.1	χ^2^ = 4.1
6000–10,000 Rs	407	53.3	343	52.0	64	62.1	df = 2
>10,000 Rs	90	11.8	82	12.4	8	7.8	*p* = 0.13
Mother’s education
Up to primary	206	27.0	181	27.4	25	24.3	χ^2^ = 0.7
Matriculation	235	30.8	200	30.3	35	34.0	df = 2
College or above	322	42.2	279	42.3	43	41.7	*p* = 0.70
Work outside the home	29	3.8	24	3.6	5	4.9	0.58
Husband’s education
Up to primary	215	28.2	190	28.8	25	24.3	χ^2^ = 1.0
Matriculation	230	30.1	196	29.7	34	33.0	df = 2
College or above	318	41.7	274	41.5	44	42.7	*p* = 0.61
Husband’s job
Unemployed	20	2.6	20	3.0	0	0	
Laborer	16	2.1	13	2.0	3	2.9	χ^2^ = 3.8
Clerical	503	65.9	436	66.1	67	65.0	df = 3
Self-employed	224	29.4	191	28.9	33	32.0	*p* = 0.28
Husband away from home for more than 1 month	71	9.3	57	8.6	14	13.6	0.14
Number of children
None	335	43.9	286	43.3	49	47.6	χ^2^ = 1.6
1–3	395	51.8	347	52.6	48	46.6	df = 2
4 Or more	33	4.3	27	4.1	6	5.8	*p* = 0.45
Ever lost a child	81	10.6	73	11.1	8	7.8	0.39
Separate family	206	27.0	179	27.1	27	26.2	0.91
Aged >25 at marriage	160	21.0	140	21.2	20	19.4	0.80
Any medication taken during pregnancy	555	72.7	476	72.1	79	76.7	0.41
Antiallergy/antibiotic/hypertensive/gyne/GI medication	48	6.3	42	6.4	6	5.8	1.0
Calcium/iron/vitamin/mineral mediation	547	71.7	469	71.1	78	75.7	0.35
Antianxiety medication	1	0.1	1	0.2	0	0	1.0
Problem with last delivery	176	23.1	151	22.9	25	24.3	0.80
Total SRQ score 7 or more at baseline	387	50.7	334	50.6	53	51.5	0.92
Total EPDS score 12 or more at baseline	580	76.0	503	76.2	77	74.8	0.80

*^a^Comparison used Fisher’s exact test for dichotomous variables, Yates’ corrected chi-squared test for other categorical variables*.

*Sig, significance*.

Using multiple logistic regression, we found no significant differences for LBW with either EPDS or SRQ scores, either unadjusted, or after adjusting for mother’s age, low education of mother or father, low father’s income or having problems with a previous delivery (Table 2).

**Table 2:** **Association between Edinburgh postnatal depression scale (EPDS) and self-reporting questionnaire (SRQ-20) scores in the third trimester of pregnancy and low-birth weight**.

	Odds ratio for each quartile increment in EPDS OR (95% CI)^a^	Odds ratio for each quartile increment in SRQ-20 OR (95% CI)^a^
Unadjusted	0.881 (0.732–1.060)	1.020 (0.845–1.231)
Adjusted for
Maternal education	0.882 (0.733–1.062)	1.024 (0.847–1.237)
Age	0.881 (0.732–1.061)	1.021 (0.845–1.232)
Income	0.881 (0.732–1.060)	1.022 (0.845–1.235)
Paternal education	0.883 (0.733–1.062)	1.025 (0.848–1.237)
Problem with previous delivery	0.877 (0.728–1.056)	1.016 (0.841–1.229)

*OR, odds ratio*.

*^a^Analyses used logistic regression*.

## Discussion

In this study, presence of depression during pregnancy was not associated with LBW in newborns. Findings of this study are similar to a recent study from Ethiopia (17), which reported no association between antenatal depression and LBW. These findings, however, contradict previous South Asian studies (14–16), which reported an association between maternal depression during antenatal period and infant’s LBW. These differences might be explainable due to the population setting. Previous studies were conducted in rural Pakistan, and it is possible that children in these studies had LBW because of mothers’ poor physical health. Health facilities are very limited in rural areas and mothers cannot easily access these facilities. Maternal education could be another potential factor. In rural areas in Pakistan, female literacy rates are very low. Low level of maternal education has been reported to be strongly associated with birth outcomes, like; preterm birth, small for gestational age (SGA) birth, still birth, and neonatal and post neonatal death (28, 29). Educated mothers are more likely to use health care services and are more likely to comply with medical advice during pregnancy than uneducated mothers. Other reasons might include methodological issues (e.g., language issues due to low level of education, biases in sample selection and even training of health workers who conducted the surveys) with previous studies.

Our study has certain strengths and limitations. Both EPDS and SRQ have been previously validated in Pakistan (20, 21). All instruments were applied by trained and experienced researchers. The major limitation of this study is very high-attrition rate. However, our sample of 763 mothers for whom we were able to obtain infant birth weights is still very large. Neither the EPDS nor the SRQ score of the mother in third trimester of pregnancy showed a significant association with low-neonatal birth weight either in univariate analyses or in any of the analyses adjusted for possible confounders, i.e., 14.2% of depressed mothers had a LBW baby compared with 13.3% of non-depressed mothers. If this study were to be repeated, then over 23,000 subjects per group would be required to have 80% to detect a significant difference between these 2 proportions using a 5% significance test. The large sample size and very small difference in proportions suggests that the absence of an association between mother’s depression and LBW is a robust finding.

We had no information regarding women who delivered outside the study center. However, the mothers with data on the baby’s birth weight were more educated, and were also more likely to have had a problem with their previous delivery compared with the rest of the groups. We adjusted for these potential confounders in our multivariate analyses and the effect on the results was negligible. It is possible that volatile law and order conditions in Karachi may be the reason for high drop-out rates. Women of this study may have delivered at their homes or nearby maternity homes during the period of unrest and poor law and order in the local area. Other limitations of our study include; not using a standard diagnostic instrument to confirm the diagnosis of depression, and collection of data from the hospital records for newborn’s birth weight. It should also be emphasized that, although the results are statistically significant, this is not the same as clinical significance.

The prevalence of antenatal depression is high in this population (25.8%), and although our study has negative findings with regard to LBW, the detection and treatment of depression in mothers during the antenatal period is very important, in order to study other adverse effects. Further research is warranted in this population, to understand both the protective and vulnerability factors associated with depression and birth outcomes.

## Author Contributions

All authors made significant intellectual contributions to this work, participated in the manuscript revision process, and approved the final manuscript for submission. In addition, the following authors participated in: Nusrat Husain and Nasim Chaudhry: Study conception, design and draft preparation, Tariq Munshi: Study design, data interpretation, and draft preparation, Farhat Jafri: Study design and execution, Meher Husain: Data analysis and interpretation, Asia Parveen: Study conception, design, and execution, Qamar Saeed: Study conception, design, and execution, Barbara Tomenson: Study conception, data analysis, and interpretation, draft preparation, Farooq Naeem: Editing, scientific writing and Grammar.

## Conflict of Interest Statement

The authors declare that the research was conducted in the absence of any commercial or financial relationships that could be construed as a potential conflict of interest.

## Abbreviations

EPDS, Edinburgh postnatal depression scale; LBW, low-birth weight; PIQ, personal information questionnaire; Rs, rupees (Pakistan currency); SRQ, self-reporting questionnaire.
